# Enrichment and identification of Δ^9^-Tetrahydrocannabinolic acid synthase from *Pichia pastoris* culture supernatants

**DOI:** 10.1016/j.dib.2015.07.033

**Published:** 2015-08-06

**Authors:** Kerstin Lange, Ansgar Poetsch, Andreas Schmid, Mattijs K. Julsing

**Affiliations:** a Laboratory of Chemical Biotechnology, Department of Biochemical & Chemical Engineering, TU Dortmund University, 44227 Dortmund, Germany; b Department of Solar Materials, Helmholtz Centre for Environmental Research (UFZ), 04318 Leipzig, Germany; c Plant Biochemistry, Ruhr University Bochum, 44801 Bochum, Germany

## Abstract

This data article refers to the report Δ^9^-Tetrahydrocannabinolic acid synthase (THCAS) production in *Pichia pastoris* enables chemical synthesis of cannabinoids (Lange et. al. 2015) [2]. THCAS was produced on a 2 L lab scale using recombinant *P. pastoris* KM71 KE1. Enrichment of THCAS as a technically pure enzyme was realized using dialysis and cationic exchange chromatography. nLC-ESI-MS/MS analysis identified THCAS in different fractions obtained by cationic exchange chromatography.

Specifications Table

**Table:** 

Subject area	Biochemistry; biocatalysis
More specific subject area	Protein isolation, protein application in synthesis and proteomics
Type of data	Gene expression data (SDS PAGE), enrichment and isolation of protein (CIEX chromatogram), Proteome discoverer search results (xls and ProtXML files), specific activities
How data was acquired	Fermentation, dialysis, CIEX, HPLC, SDS PAGE, one- dimensional nLC-ESI-MS/MS
Data format	analyzed
Experimental factors	THCAS was produced with *P. pastoris* KM71 KE1; activity was determined; bands of protein gel were analyzed
Experimental features	THCAS is secreted in two active fractions with differences in glycosylation patterns
Data source location	N/A
Data accessibility	Data is provided as Supplementary material directly with this article, data are also related to Lange et al [2]

Value of the data

• Heterologous production of plant proteins in *P. pastoris* benefits from glycosylation, but one should be aware it might result in a mixture of proteins with different glycosylation patterns and thus different enzymatic properties.
• nLC-ESI-MS/MS is an efficient method to detect and identify low amounts of recombinant protein
• The simple two-step protocol for THCAS enrichment might also be applied for the fast isolation of other positively charged proteins from *P. pastoris* culture supernatants.
• Chemical synthesis of complex and hydrophobic natural products can benefit from the implementation of biocatalytic reactions derived from the natural source.

## 1 Data, experimental design, materials and methods

### 1.1 Cloning

**Fig. 1:**
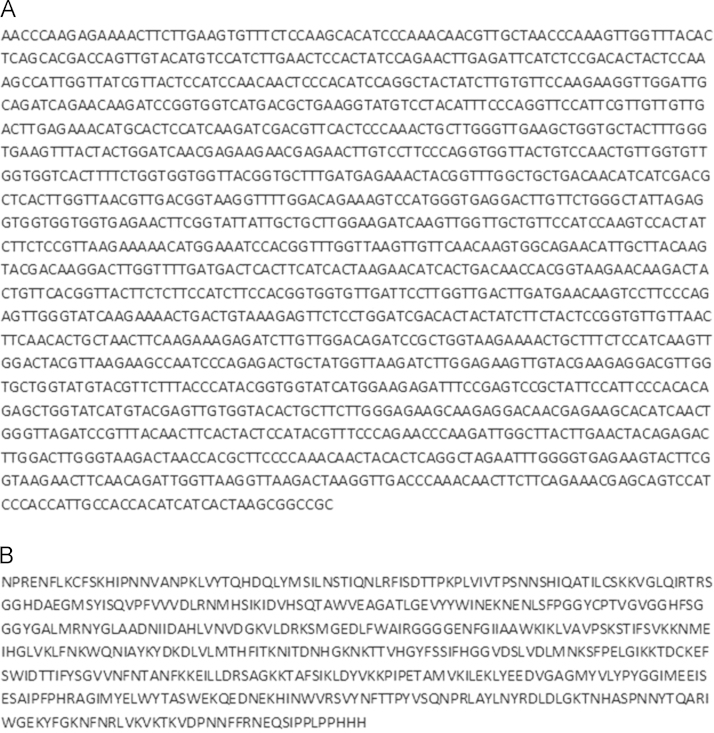
(A) Codon optimized gene sequence (GenBank accession no. AB057805) of *thcas* gene cloned into pPICZαA plasmid using *Eco*RI and *Not*I restriction sites; the N-terminal signal sequence was removed from the gene sequence and replaced by the α- mating factor signal peptide (sequence encoded on plasmid); (B) amino acid sequence of THCAS.

A codon optimized gene sequence of the *thcas* gene (Fig. 1) was cloned into the plasmid pPICZαA (Life Technologies GmbH, Darmstadt, Germany) using *Eco*RI and *Not*I restriction sites. The native N-terminal signal sequence of *thcas* was removed and replaced by the α-mating factor signal peptide of *Saccharomyces cerevisiae* (encoded on plasmid). *P. pastoris* Mut^S^ strain KM71 was transformed with 1 µg of the *Sac*I linearized plasmid according to existing protocols resulting in the strain *P. pastoris* KM71 KE1 [1]. Afterwards cells were plated on YPDS agar plates containing zeocin (100 µg mL^−1^). The cells were cultivated in BMGY medium and BMMY medium and activity was tested in cell free culture supernatant, in order to identify colonies of *Pichia pastoris*, which were able to secrete active THCAS.

### 1.2 Media

All chemicals were obtained from SigmaAldrich (Steinheim, Germany), Carl Roth GmbH & Co KG (Karlsruhe, Germany), AppliChem GmbH (Darmstadt, Germany) or Merck KGaA (Darmstadt, Germany) in the highest purity available.

The following media were used for transformation of the *P. pastoris* strain: YPD (Yeast Extract peptone Dextrose Medium): 10 g yeast extract, 20 g peptone, 20 g glucose. For growth on solid medium, 15 g L^−1^ agar-agar was added to the medium. YPDS: the same composition as YPD medium, but with additional 182.2 g of sorbitol per 20 g of agar.

For shaking flask cultivation buffered glycerol-complex medium (BMGY) and buffered methanol-complex medium (BMMY) were used. The compositions of the media were as follows (per liter): BMGY: 10 g yeast extract, 20 g/L peptone, 1.34 g yeast nitrogen base without amino acids, 0.4 mg biotin, 10 mL glycerol, 100 mL 1 M potassium phosphate buffer (pH 6.0); BMMY: identical to BMGY medium, but glycerol was exchanged to 0.5% methanol (v/v).

For the cultivation in the bioreactor experiments basal salt medium was used with following composition (per liter): 1.18 g calcium sulfate×2H_2_O, 18.2 g potassium sulfate, 14.9 magnesium sulfate×7H_2_O, 4.13 g potassium hydroxide, 40 g glycerol, 26.7 mL phosphoric acid (85%). PTM_1_ trace salts were used for complementation of basal salt medium (4.35 mL per liter of fermentation basal salts medium) in following composition (per liter): 6 g cupric sulfate×5H_2_O, 0.08 g sodium iodide, 3 g manganese sulfate, 0.2 g sodium molybdate×2H_2_O, 0.02 g boric acid, 0.5 g cobalt chloride, 20 g zinc chloride, 65 g ferrous sulfate 7H_2_O, 0.2 g biotin, 5 mL sulfuric acid.

### 1.3 Cultivation of *Pichia pastoris*

**Fig. 2:**
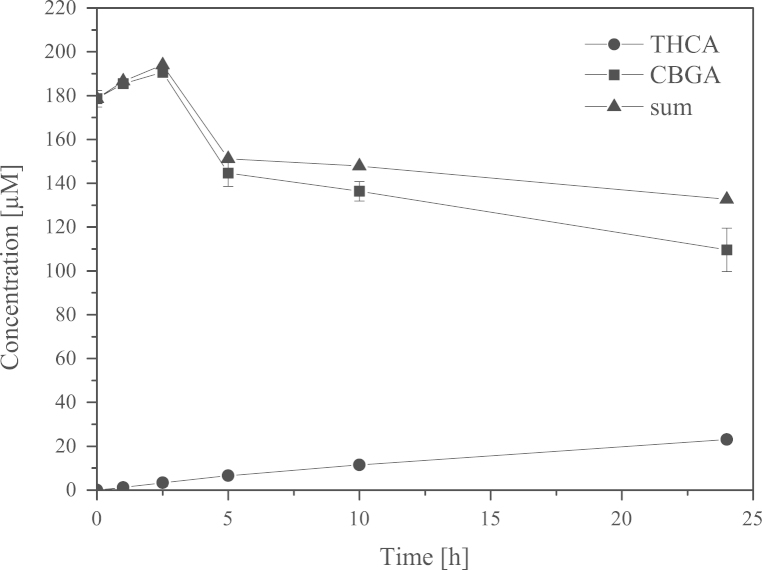
Conversion of CBGA into THCA by culture supernatant of *P. pastoris* KM71 KE1; CBGA was added to the culture supernatant and the amounts of CBGA and THCA were measured with HPLC as described in Lange et al. [2]; measurements are means of duplicates; standard deviation was always within 5%.

*P. pastoris* KM71 KE1 was cultivated in BMGY medium overnight for small scale expression (50 mL in baffled shaking flask, 30 °C, 200 rpm). After centrifugation (4700 rpm, 10 min, 4 °C) the cells were resuspended in BMMY medium (20 mL) and transferred to baffled shaking flasks containing 80 mL BMMY medium. Cultivation was performed for 3 days at 20 °C and 100 rpm. We detected activity by measuring formation of Δ^9^-tetrahydrocannabinolic acid (THCA) from cannabigerolic acid (CBGA) in the cell free culture supernatant (Fig. 2).

**Fig. 3:**
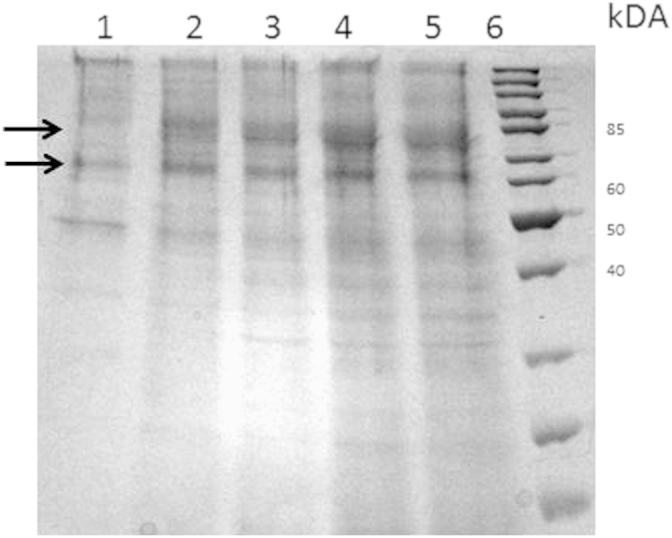
Protein secretion over the fermentation of *Pichia pastoris* KM71 KE1 producing THCAS; lane 1: before induction; lane 2: 24 h after induction; lane 3: 47 h after induction; lane 4: 73 h after induction; lane 5: 93 h after induction; lane 6: molecular weight marker (# 26614, Thermo Fisher Scientific, Braunschweig; Germany); arrows indicate bands of THCAS.

**Fig. 4:**
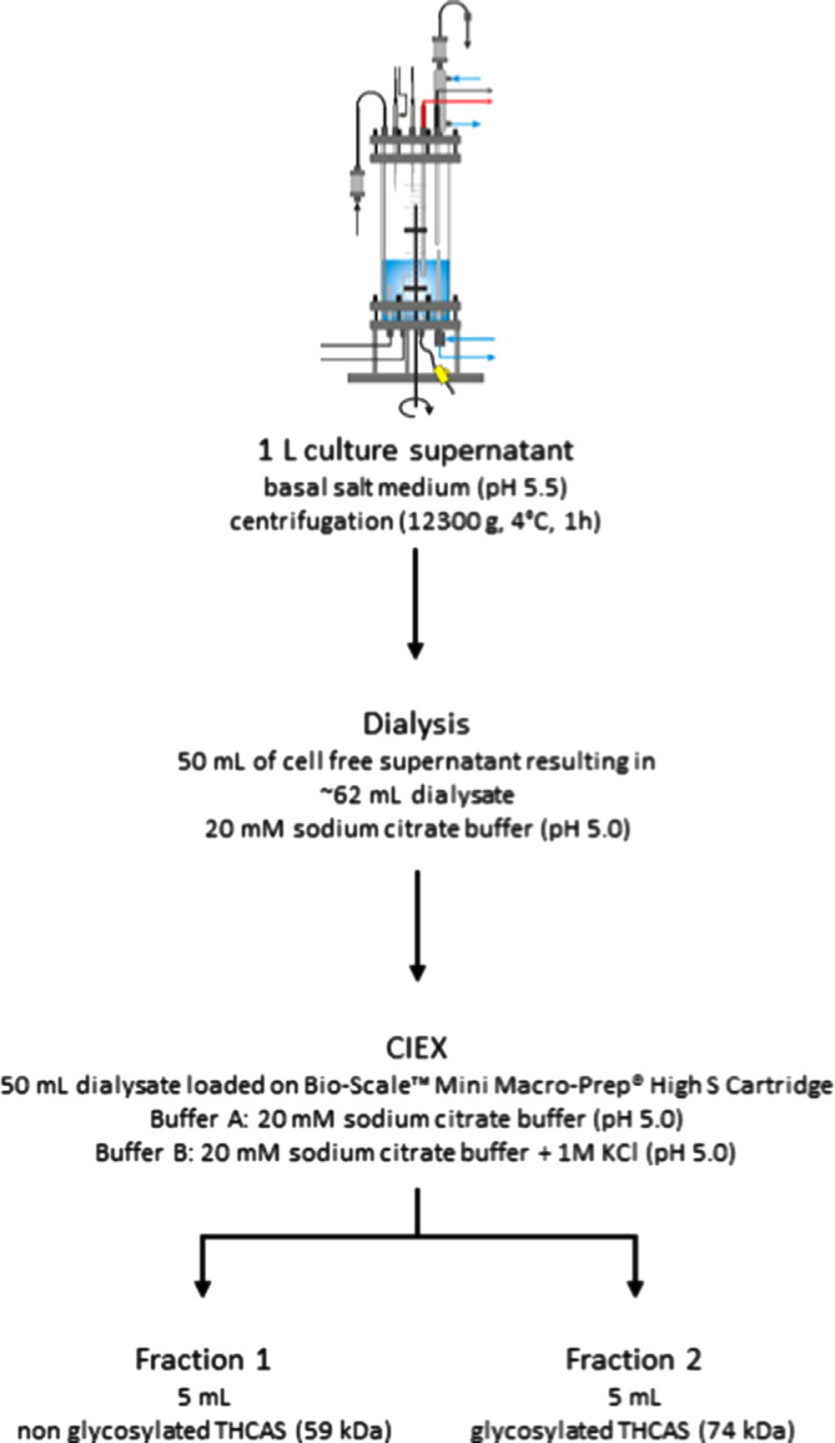
Scheme of the enrichment/isolation of THCAS from culture supernatant. Activity assays were performed with culture supernatant, dialysate, and active fractions 1 and 2. The two active fractions differ in activity resulting from two different conformations of the protein. Active fraction 1 contains mainly nonglycosylated THCAS, active fraction 2 contains glycosylated THCAS.

Cultivation of *P. pastoris* KM71 KE1 on a technical lab scale was performed in a 2 L stirred tank bioreactor KLF2000 (Bioengineering AG, Wald, Switzerland) as described in [2]. During the fermentation, secreted protein was monitored by SDS PAGE (Fig. 3) indicating two bands of THCAS in the culture supernatant (~59 kDa non-glycosylated and ~74 kDa glycosylated). After the fermentation, cells were removed by centrifugation (12,300*g*, 4 °C, 1 h) in order to obtain the culture supernatant containing active THCAS. Supernatant was further used for activity assays or for the enrichment of THCAS. (Fig. 4)

### 1.4 Enrichment/isolation of THCAS

**Fig. 5:**
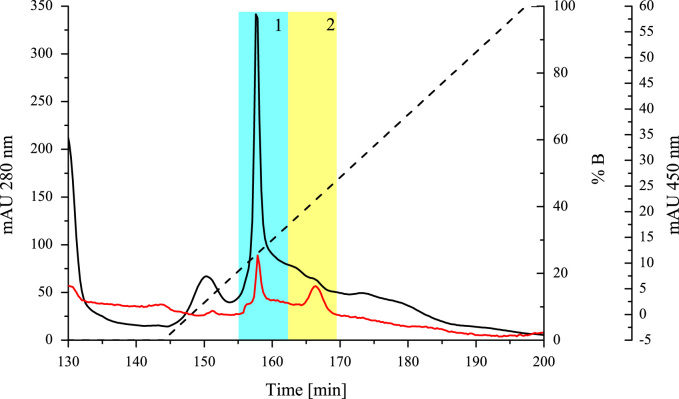
Chromatogram of cationic exchange chromatography; the chromatogram indicates the protein content in the eluate and the concentration of buffer B in the elution profile (gradient elution from 0 to 1 M KCl over 5 CV); UV 280 signal is represented by the black trace; the UV 450 signal (red trace) increasing at 155 min also indicates the presence of flavoprotein; active THCAS could be found in fraction 1 (blue box) with a size of 59 kDa and in fraction 2 (yellow box) with a size of 74 kDa.

A total of 1 L culture supernatant could be obtained via lab scale fermentation. Enrichment of THCAS was performed stepwise from this liter (Fig. 4) 50 mL of the culture supernatant was dialyzed against 5 L of 20 mM sodium citrate buffer at 4 °C using a ZelluTrans membrane (MWCO 6000–8000, Carl Roth, Karlsruhe, Germany). The buffer was exchanged twice. 50 mL of the obtained dialysate (~62–65 mL) were loaded on a Bio-Scale™ Mini Macro-Prep^®^ High S Cartridge (5 mL, Bio-Rad, München, Germany) for subsequent cationic exchange chromatography. The column was connected to an Äkta purifier™ chromatography system (GE Healthcare, Freiburg, Germany). The column was equilibrated with 5 column volumes of buffer A (20 mM sodium citrate buffer, pH 5.0). For the elution of THCAS and other proteins, a linear gradient was applied ranging from 0 to 100% of buffer B (20 mM sodium citrate buffer including 1 M potassium chloride, pH 5.0). Cationic exchange chromatography resulted in the elution of two active fractions (Fig. 5). Fig. 7 shows the two active fractions before and after treatment with EndoH. All steps were carried out at 4 °C. 

### 1.5 Activity tests

**Fig. 6:**
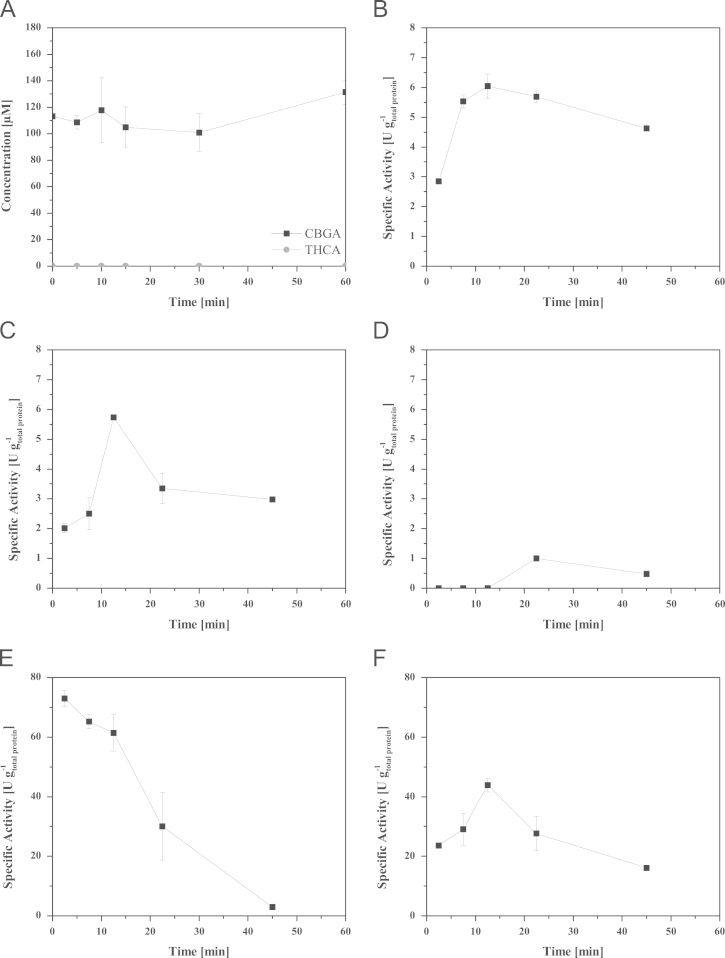
Specific activities over time from fermentation over dialysis and CIEX; (A) substrate concentration in control sample (cell free culture supernatant before induction); as no activity/product formation of THCA could be observed, the activity is zero before induction; activity was determined with cell free culture supernatant in basal salt medium (pH 5.5) (B) specific activity in culture supernatant after end of the fermentation in basal salt medium (pH 5.5); (C) specific activity in culture supernatant after dialysis in 20 mM sodium citrate buffer (pH 5.0); (D) specific activity in flowtrough of CIEX step in 20 mM sodium citrate buffer (pH 5.5); (E) specific activity of active fraction 1 (59 kDa) in 20 mM sodium citrate buffer containing 28 mM KCl (pH 5.5); (F) specific activity of active fraction 2 (74 kDa) in 20 mM sodium citrate buffer containing 40 mM KCl (pH 5.5); specific activity was determined based on duplicate assays; specific activity was calculated based on total protein amount in the respective fraction, which were determined based on the method of Bradford.

**Fig. 7:**
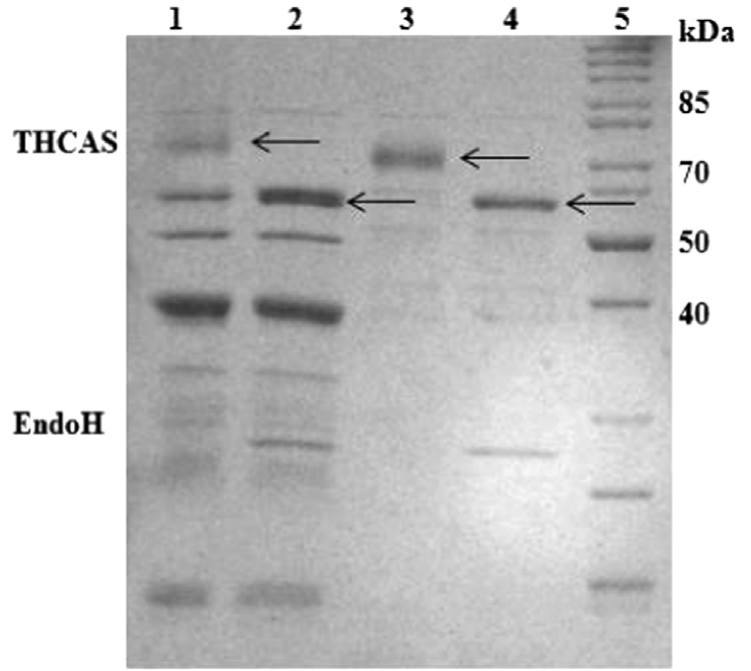
SDS-Page of the two active fractions containing THCAS before and after treatment with Endo H; lane 1: active fraction 1 before EndoH treatment; lane 2: active fraction 1 after EndoH treatment; lane 3: active fraction 2 before EndoH treatment; lane 4: active fraction 2 after EndoH treatment; lane 5: molecular weight marker (#26614, Thermo Fisher Scientific, Braunschweig; Germany); THCAS can be found at 59 kDa and 74 kDa; a band of EndoH can be found in lane 2 and lane 4 between 25 and 30 kDa; samples were concentrated 25 fold for SDS PAGE using 1.5 mL centrifugal devices with a molecular weight cut off of 10 kD (membrane of polyether sulfone, VWR, Darmstadt, Germany); arrows indicate the bands, which were identified as THCAS by nLC-ESI-MS/MS analysis (see Supplementary information).

Activity tests were performed with samples of culture supernatant obtained from shaking flask experiments (BMMY medium, pH 6.0), bioreactor experiments (basal salt medium, pH 5.5) or with dialysate (20 mM sodium citrate buffer, pH 5.0) and active fractions (20 mM sodium citrate buffer, pH 5.0 containing 28 mM KCl in fraction 1 and 40 mM KCl in fraction 2). Activity assays (Fig. 6) were performed in 100 µL total volume. The assay was started by the addition of 150 µM CBGA (from a 10 mM stock solution, CBGA dissolved in MeOH) to the assay. The tubes were incubated at 30 °C and 600 rpm in a thermoshaker for 0, 5, 10, 15, 30 and 60 min (Eppendorf, Hamburg, Germany). The assay was stopped by the addition of 100 µL pure MeOH to the respective sample. After extensive mixing and centrifugation (10 min, 4700 rpm, 4 °C), 50 µL of the respective sample was analyzed by HPLC. The reader is referred to the associated research article [2] for a detailed description of the HPLC analysis method. Specific activity was calculated based on the total protein amounts in the respective sample determined by the method of Bradford [3]. Specific activities were calculated as U g^-1^_total protein_ (1 U equals the formation of 1 µmol THCAS per minute). For the specific activity during course of the assay the product concentration was subtracted from the concentration at following time point resulting in new generated product concentration between the two time points.

### 1.6 In-gel tryptic digestion

Protein bands were excised from the gels, cut into small cubes (ca. 1×1 mm^2^), and destained according to Schlüsener and colleagues [4]. Gel pieces were dried in a SpeedVac, trypsin (porcine, sequencing grade; Promega, Mannheim, Germany) solution (12.5 ng ml^−1^ in 25 mM ammonium bicarbonate, pH 8.6) was added until gel pieces were immersed completely in digestion solution. The protein digestion was performed over night at 37 °C with agitation (tempered shaker HLC MHR20, 550 rpm). After digestion, elution buffer (50% acetonitrile, 0.5% TFA, UPLC grade, Biosolve, Netherlands) was added (1 µl elution buffer for each µl of digestion buffer) and the samples were sonicated for 20 min in an ultrasonic bath. Samples were centrifuged and supernatants were transferred to new 1.5 ml tubes. The extracted peptides were dried using a SpeedVac and stored at −20 °C. Prior to MS-analysis peptides were resuspended in 20 µl of buffer A (0.1% formic acid in water, ULC/MS, Biosolve, Netherlands) by sonication for 10 min and transferred to LC-MS grade glass vials (12×32 mm^2^ glass screw neck vial, Waters, USA). Each measurement was performed with 8 μL of sample.

### 1.7 One-dimensional nLC-ESI-MS/MS

An UPLC HSS T3 column (1.8 µm, 75 µm×150 mm, Waters, Milford, MA, USA) and an UPLC Symmetry C_18_ trapping column (5 µm, 180 µm×20 mm, Waters, Milford, MA, USA) for LC as well as a PicoTip Emitter (SilicaTip, 10 µm i.d., New Objective, Woburn, MA, USA) were used in combination with the nanoACQUITY gradient UPLC pump system (Waters, Milford, MA, USA) coupled to a LTQ Orbitrap Elite mass spectrometer (Thermo Fisher Scientific Inc., Waltham, MA, USA). For elution of the peptides a gradient with increasing concentration of buffer B (0.1% formic acid in acetonitrile, ULC/MS, Biosolve, Netherlands) was used in 105 min at a flow rate of 400 nL min^−1^ and a spray voltage of 1.6 kV: 0–5 min: 2% buffer B; 5–10 min: 2–5% buffer B; 10–71 min: 5–30% buffer B; 72–77 min: 85% buffer B; 77–105 min: 2% buffer B. The analytical column oven was set to 55 °C and the heated desolvation capillary was set to 275 °C. The LTQ Orbitrap Elite was operated via instrument method files of Xcalibur (Rev. 2.1.0) in positive ion mode. The linear ion trap and Orbitrap were operated in parallel, i.e. during a full MS scan on the Orbitrap in the range of 150–2000 *m/z* at a resolution of 60,000 MS/MS spectra of the 20 most intense precursors were detected in the ion trap using the rapid scan mode. The relative collision energy for collision-induced dissociation (CID) was set to 35%. Dynamic exclusion was enabled with a repeat count of 1 and 45 s exclusion duration window. Singly charged and ions of unknown charge state were rejected from MS/MS.

### 1.8 Protein identification

Proteins were identified using the SEQUEST [5] algorithm embedded in Proteome Discoverer 1.4 (Thermo Electron^©^ 2008–2012) searching against the complete proteome database of *Komagataella* (*Pichia*) *pastoris* (strain GS115 / ATCC 20864) containing 5073 entries obtained from UniProt (UP000000314), additionally including the protein sequence of THCAS. The mass tolerance for precursor ions was set to 10 ppm; the mass tolerance for fragment ions was set to 0.6 Da. Only tryptic peptides with up to two missed cleavages were accepted and the oxidation of methionine was admitted as a variable peptide modification. The false discovery rate (FDR) was determined with the percolator validation in Proteome Discoverer 1.4 and the *q*-value was set to 1% [6]. For protein identification the mass spec format-(msf)-files were filtered with peptide confidence “high” and two unique peptides per protein. Results were exported from Proteome Discoverer as Excel tables (Supplementary information S1-1 to S4-1) and in ProtXML files (S1-2 to S4-2) format.

## Funding

This project was supported by funds from the Ministry of Innovation, Science and Research of North Rhine-Westphalia in the frame of CLIB-Graduate Cluster Industrial Biotechnology, contract no: 314-108 001 08.

## Footnotes

Appendix A Supplementary data associated with this article can be found in the online version at doi:10.1016/j.dib.2015.07.033.
